# Obesity is associated with severe COVID-19 but not death: a dose−response meta-analysis

**DOI:** 10.1017/S0950268820003179

**Published:** 2021-01-05

**Authors:** Linyan Deng, Jiaoyue Zhang, Mengyuan Wang, Lulu Chen

**Affiliations:** 1 Department of Endocrinology, Union Hospital, Tongji Medical College, Huazhong University of Science and Technology, Wuhan, Hubei 430022, China; 2 Hubei provincial Clinical Research Center for Diabetes and Metabolic Disorders, Wuhan, Hubei 430022, China

**Keywords:** COVID-19, meta-analysis, mortality, obesity, severity

## Abstract

The coronavirus disease 2019 (COVID-19) epidemic is spreading globally. Studies revealed that obesity may affect the progression and prognosis of COVID-19 patients. The aim of the meta-analysis is to identify the prevalence and impact of obesity on COVID-19. Studies on obese COVID-19 patients were obtained by searching PubMed, Cochrane Library databases and Web of Science databases, up to date to 5 June 2020. And the prevalence rate and the odds ratio (OR) of obesity with 95% confidence interval (CI) were used as comprehensive indicators for analysis using a random-effects model. A total of 6081 patients in 11 studies were included. The prevalence of obesity in patients with COVID-19 was 30% (95% CI 21–39%). Obese patients were 1.79 times more likely to develop severe COVID-19 than non-obese patients (OR 1.79, 95% CI 1.52–2.11, *P* < 0.0001, *I*^2^ = 0%). However obesity was not associated with death in COVID-19 patients (OR 1.05, 95% CI 0.65–1.71, *P* = 0.84, *I*^2^ = 66.6%). In dose−response analysis, it was estimated that COVID-19 patients had a 16% increased risk of invasive mechanical ventilation (OR 1.16, 95% CI 1.10–1.23, *P* < 0.0001) and a 20% increased risk of admission to ICU (OR 1.20, 95% CI 1.11–1.30, *P* < 0.0001) per 5 kg/m^2^ increase in BMI. In conclusion, obesity in COVID-19 patients is associated with severity, but not mortality.

## Introduction

Severe acute respiratory syndrome coronavirus 2 (SARS-CoV-2) causes coronavirus disease 2019 (COVID-19) and poses a serious threat to world public health security [1–3]. COVID-19 spreads rapidly and has a high mortality rate. As of 31 July 2020, the World Health Organization (WHO) reported that there had been 10 185 374 confirmed cases and 503 862 deaths worldwide.

Studies have shown that hypertension, diabetes, respiratory diseases and cardiovascular diseases are risk factors for COVID-19 patients [4, 5]. The prevalence of obesity has increased exponentially over the last 30 years. Obesity is associated with an increased risk of cardiovascular disease and diabetes [6, 7]. However, the association between obesity and COVID-19 has rarely been reported in early clinical studies. Previous research studies have revealed that obesity increases the risk of some infections [8–10]. Moreover, obesity is associated with poor prognosis in influenza A (H1N1) patients [11]. Recent investigations have reported that obesity might increase the risk of SARS-CoV-2 infection and affect the prognosis of patients with COVID-19 [12, 13]. Whereas some cohort studies with COVID-19 disease found obesity rates were generally reported as no higher than population-based estimates [14, 15]. A recent Italian report failed to mention obesity as a co-morbidity in admitted COVID-19 intensive care unit (ICU) patients [16]. Yang *et al*. [17] reported that severe COVID-19 patients had higher body mass index (BMI) than non-severe ones, and COVID-19 patients with obesity were more severe and have a worse outcome than those without. However, mortality was not included in their meta-analysis. Emerging data support a survival benefit of obesity in critical illness, and the phenomenon has been coined ‘the obesity survival paradox’ [18, 19]. Therefore, it remains controversial whether obesity is related to increased susceptibility and adverse outcomes among patients with COVID-19 owing to the heterogeneous source of the existing cohort data.

To clarify the relationship between obesity and COVID-19, two existing public health epidemics, we conducted a meta-analysis to explore the prevalence of obesity in patients with COVID-19 and the relationship between obesity and the severity and mortality of COVID-19.

## Methods

### Search strategy and study selection

The meta-analysis was performed according to the Preferred Reporting Items for Systematic reviews and Meta-Analyses (PRISMA) [20]. (Supplementary file 1)

We searched PubMed, Cochrane Library databases and Web of Science databases carefully, up to date to 5 June 2020. The following search terms were used in our search strategy: ‘COVID-19’, ‘SARS-CoV-2’, ‘2019-nCoV’, ‘coronavirus disease-19’, ‘new coronavirus’, ‘novel corona virus’, ‘novel coronavirus’, ‘nCoV-2019’, ‘2019 novel coronavirus’, ‘coronavirus disease 2019’ or ‘severe acute respiratory syndrome coronavirus 2’ and ‘obesity’ or ‘BMI’. And we screened the references of the articles assessed for eligibility to identified additional records.

Inclusion criteria: (1) Participants: patients diagnosed with SARS-CoV-2 infection; (2) Intervention: COVID-19 patients with obesity included BMI data; (3) Outcome: the prevalence and the odds ratio (OR) of obesity with 95% confidence interval (CI); (4) Study: Observational studies such as cohort studies on COVID-19 and obesity, which were written in English language.

Exclusion criteria: (1) Review, case report, comment, editorial; (2) Only children, pregnant women and COVID-19 patients with other complications have been studied; (3) The study did not classify the severity or mortality of COVID-19 patients; (4) Insufficient data information is provided.

### Data extraction and quality assessment

We extracted the data in the selected articles as the following: first author, date, country, number of patients, male to female ratio, median age, obesity definition, outcome (basis of disease severity or death), proportion of obesity in severe and non-severe patient (or proportion of obesity in deaths and survivors), quality assessment and the prevalence of obesity, hypertension, diabetes mellitus, smoker and chronic obstructive pulmonary diseases etc. In addition, we collected the number of COVID-19 cases and the number of severe COVID-19 cases and deaths at different BMI levels.

Newcastle-Ottawa quality assessment scale (NOS) was used to assess the quality of cohort study [21]. The qualities of the studies were classified as low (<4), moderate (4–6) and high (>6).

### Data analysis

STATA MP version 16.0 was used to analyse the data. We used forest plots to describe the prevalence and impact of obesity on COVID-19. And the prevalence and the OR of obesity with 95% CI were the principal summary measures. The data were combined using a random-effects model. Using the fixed-effect model and based on the generalised least-squares method (‘glst’ command), the dose−response analysis was realised, and the OR per 5-unit increase in the BMI levels for each study was calculated and the dose−response relationship curve was plotted. The midpoints of the upper and lower limits of each BMI category were extracted as the point estimates of the group. If the lowest or highest BMI category is an open interval, we assume that the length of the open interval is twice the length of the adjacent interval. *I*^2^ statistics was used to assess statistical heterogeneity and the *p*-value was used to determine if the result is significant statistically. When *I*^2^ ≥ 50%, it indicated the existence of obvious heterogeneity. The results were statistically significant when *P* < 0.05.

The studies were grouped by country and obesity definition. And these studies had two outcomes, whether the patient survived or whether the disease was severe. In addition, because each study determined the severity of COVID-19 differently, these studies were divided into two subgroups based on whether patients were admitted to the ICU, whether invasive mechanical ventilation (IMV) was required. And we use the funnel plots to assess the publication bias.

## Results

### Study selection

**Fig. 1.**
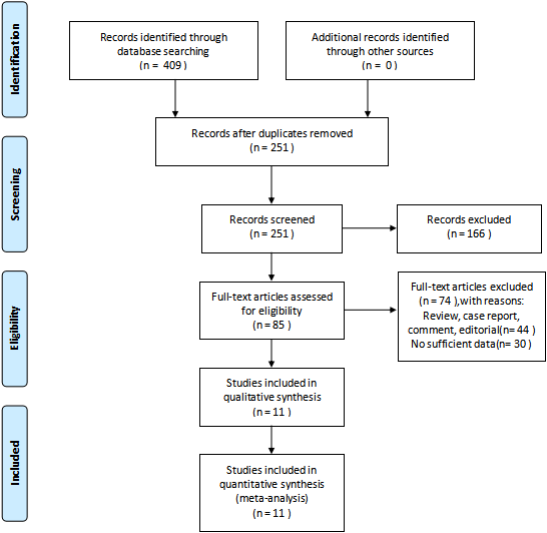
Flow diagram of study selection.

The process of study selection is shown in Figure 1. A total of 409 studies were identified from PubMed, Cochrane Library databases and Web of Science databases. In all, there were 251 studies after duplicates were removed. After reviewing the titles and abstracts, 85 studies were left to be read in full to assess their eligibility. Finally, 11 studies [15, 22–31] met the inclusion criteria for meta-analysis.

### Study characteristics

A total of 6081 patients in 11 studies were included in the meta-analysis. And the prevalence of obesity in COVID-19 patients was reported in all 11 studies. Nine studies reported the association between obesity and risk of severe SARS-CoV-2 infection [15, 22–29]. Two studies only determined the severity of COVID-19 based on the need for IMV [22, 23]. Two studies only determined the severity of COVID-19 based on whether patients were admitted to the ICU [24, 25]. Seven studies reported the association between obesity and mortality [15, 26–31]. These studies used NOS to evaluate quality, all of which were of high quality. More details are shown in Table 1.

**Table 1.** Characteristics of the 10 studies included in the meta-analysis

First author	Date	Country	Sample size (*n*)	Sex (M/F)	Median Age (years)	HTN	DM	Smoker	COPD	Obesity (%)	Obesity definition（BMI, kg/m^2^)	Basis of disease severity	Obesity /severe	Obesity/not-severe	Quality assessment
Hur, K. *et al*. [22]	2020.05.19	USA	486	271/215	59	54.9%	32.9%	33.5%	NR	259 (53.3%)	≥30	IMV	83/138	176/348	High
Toussie, D. *et al*. [23].	2020.05.14	USA	145	104/41	40	22.1	13.8	NR	NR	80 (55.2%)	≥30	IMV	20/28	60/117	High
Kalligeros, M. *et al*. [24]	2020.04.30	USA	103	63/40	60	64%	36.8%	46.6%	NR	49 (47.6%)	≥30	ICU admission	25/44	24/59	High
Urra, J.M. *et al*. [25]	2020.05.29	Europe (Spain)	172	104/68	NR	50.6%	22.7%	NR	9.9%	17 (9.9%)	≥30	ICU admission	7/27	10/145	High
Cai, Q. *et al*. [15]	2020.04.01	China	383	184/199	NR	15.1%	5.7%	NR	8.36%	41 (10.7%)	≥28	IMV	5/35	36/348	High
												ICU admission	5/35	36/348	High
												Death	1/3	40/380	High
Busetto, L. *et al*. [26]	2020.05.28	Europe (Italy)	92	57/35	70.5	64.1%	30.4%	NR	NR	29 (31.5%)	≥30	IMV	2/9	27/83	High
												ICU admission	7/16	22/76	High
												Death	2/12	27/80	High
Hajifathalian, K. *et al*. [27]	2020.05.29	USA	770	468/302	63.5	56.1%	30.9%	NR	NR	277 (36%)	≥30	IMV	91/193	186/577	High
												ICU admission	92/196	185/574	High
												Death	22/88	255/682	High
Palaiodimos, L. *et al*. [28]	2020.05.16	USA	200	98/102	64	76%	39.5%	32.5%	14%	46 (23%)	≥35	IMV	16/42	30/158	High
												ICU admission	11/32	35/168	High
												Death	16/48	30/152	High
Ong, S.W.X. *et al*. [29]*	2020.05.08	Singapore	91	51/40	NR	33%	19.8%	4.4%	NR	11 (12.1%)	≥30	IMV	8/16	32/75	High
												ICU admission	12/27	28/64	High
												Death	1/4	39/87	High
Giacomelli, A. *et al*. [30]	2020.05.21	Europe (Italy)	233	161/72	61	NR	NR	30%	NR	38 (16.3%)	≥30	Death	13/48	25/185	High
Klang, E. *et al*. [31]	2020.05.23	USA	3406	1961/1445	NR	67.5%	46.9%	23.3%	NR	1231 (36.1%)	≥30	Death	384/1136	847/2270	High

HTN: hypertension, DM: diabetes mellitus, COPD: chronic obstructive pulmonary diseases, BMI: body mass index, ICU: intensive care unit, IMV: invasive mechanical ventilation.

*Different outcomes for severity of COVID-19 with BMI≥25 kg/m^2^ and BMI<25 kg/m^2^

Six, five and four studies reported the number of COVID-19 patients using IMV, entering ICU and dying at different BMI levels. These studies were incorporated into the dose−response analysis. More details are shown in Tables S1–S3.

### Prevalence of obesity

**Fig. 2.**
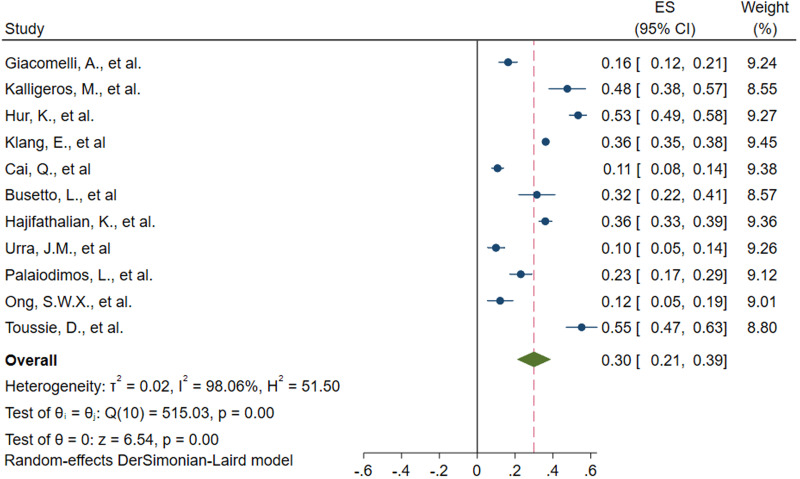
Forest plot of studies about prevalence of obesity in patients with COVID-19.

The meta-analysis of 11 studies showed that the prevalence of obesity in patients with COVID-19 was 30% (95% CI 21–39%) (Fig. 2). Among patients with COVID-19, the prevalence of hypertension was 50% (95% CI 37–64%), diabetes was 28% (95% CI 16–40%), smoker was 28% (95% CI 19–37%) and chronic obstructive pulmonary diseases was 10% (95% CI 7–14%) (Figs S1–S4).

The 11 studies were subgroup analysed by different countries and obesity definitions. The prevalence of obesity in patients with COVID-19 in USA 41.4% (95% CI 33.9–49%) was higher than in Asia 10.9% (95% CI 8.1–13.8%) and Europe 18.3% (95% CI 8.6–28%). This may be due to the higher prevalence of obesity in USA. The prevalence of obesity in patients with COVID-19 was 32.9% (95% CI 23.7–42.1%), when obesity is defined as BMI greater than or equal to 30 kg/m^2^. More information is shown in Table S4.

### Risk for severe COVID-19

**Fig. 3.**
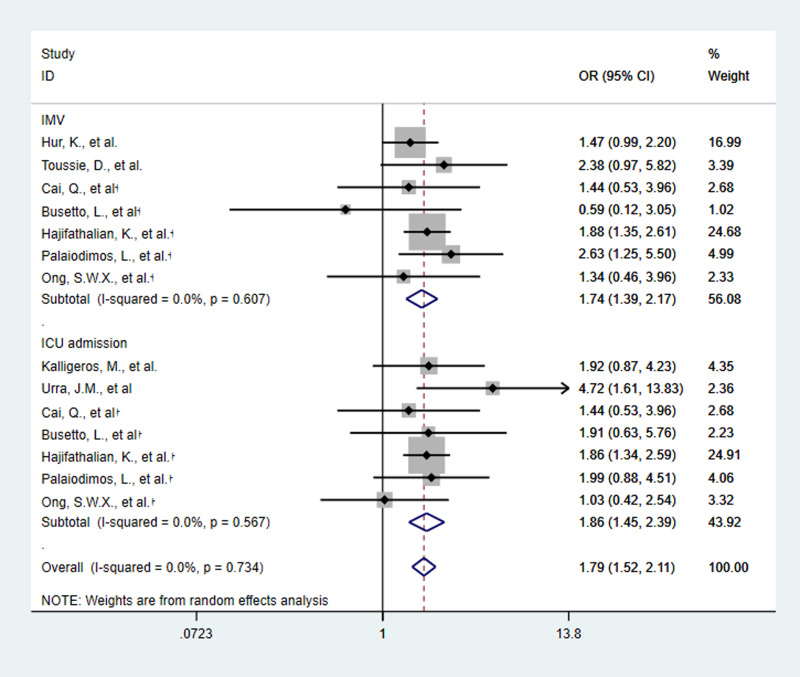
Forest plot of studies about association between obesity and risk of severe SARS-CoV-2 infection, subgroup analysed by basis of disease severity. †:basis of disease severity was ICU admission †:basis of disease severity was IMV.

**Fig. 4.**
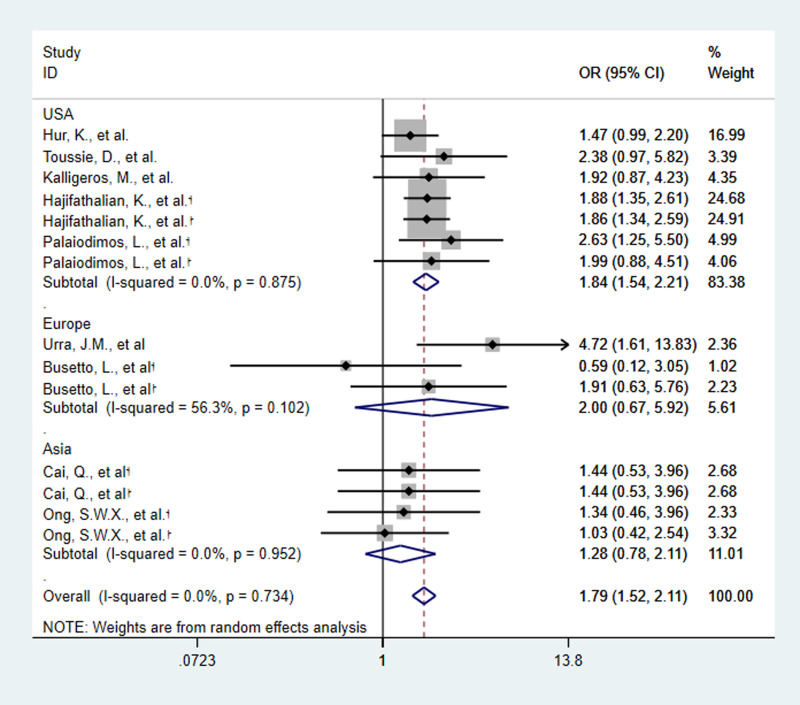
Forest plot of studies about association between obesity and risk of severe SARS-CoV-2 infection, subgroup analysed by country. †:basis of disease severity was ICU admission †:basis of disease severity was IMV.

**Fig. 5.**
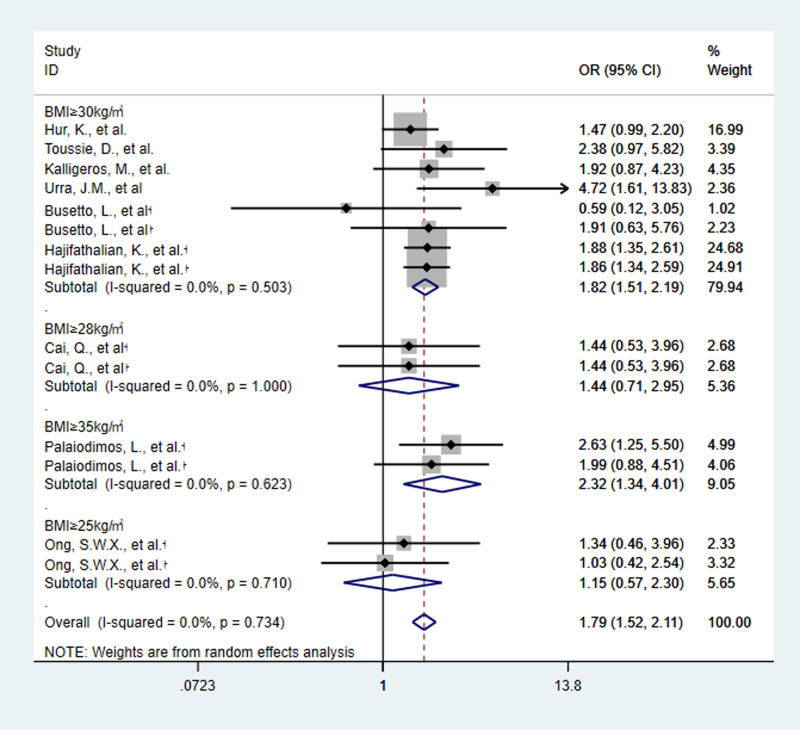
Forest plot of studies about association between obesity and risk of severe SARS-CoV-2 infection, subgroup analysed by obesity definition. †:basis of disease severity was ICU admission †:basis of disease severity was IMV..

Five of the nine studies provided two criteria (ICU admission and IMV) for determining the severity of COVID-19. Then, 14 estimates of the nine included studies showed the relationship between obesity and the severity of COVID-19. The meta-analysis of nine studies involving 2442 patients showed that obesity increased the risk of severe SARS-CoV-2 infection. Obese patients are 1.79 times more likely to develop severe COVID-19 than non-obese patients (OR 1.79, 95% CI 1.52–2.11, *P* < 0.0001, *I*^2^ = 0%) (Figs 3–5).

The nine studies were subgroup analysed by basis of disease severity, country and obesity definition. ICU admission and IMV as the criterion for determining the severity of COVID-19, OR was as the following: 1.86 (95% CI 1.45–2.39, *P* < 0.0001, *I*^2^ = 0%) and 1.74 (95% CI 1.39–2.17, *P* < 0.0001, *I*^2^ = 0%) (Fig. 3). The results revealed that obesity increased the risk of ICU admission and IMV use in COVID-19 patients.

Subgroup analysis by country showed that OR in Europe was 2.00 (95% CI 0.67–5.92, *P* = 0.21, *I*^2^ = 56.3%), in USA was 1.84 (95% CI 1.54–2.21, *P* < 0.0001, *I*^2^ = 0%) and in Asia was 1.28 (95% CI 0.78–2.11, *P* = 0.32, *I*^2^ = 0%) (Fig. 4). The results revealed that obesity increased the risk of severe SARS-CoV-2 infection in USA. And when obesity is defined as BMI of 30 kg/m^2^ or greater, OR was 1.82 (95% CI 1.51–2.19, *P* < 0.0001, *I*^2^ = 0%) (Fig. 5). It means that the risk of severe COVID-19 infection is greater, when BMI is 30 kg/m^2^ or greater.

**Fig. 6.**
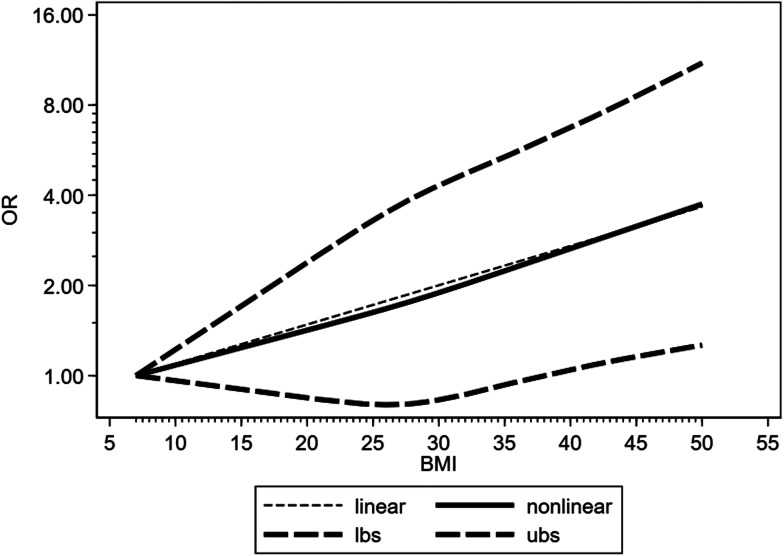
Dose−response relationship between BMI and risk of IMV using for COVID-19 patients. Long-dashed black lines represent 95% CI. The vertical axes are on a log scale.

**Fig. 7.**
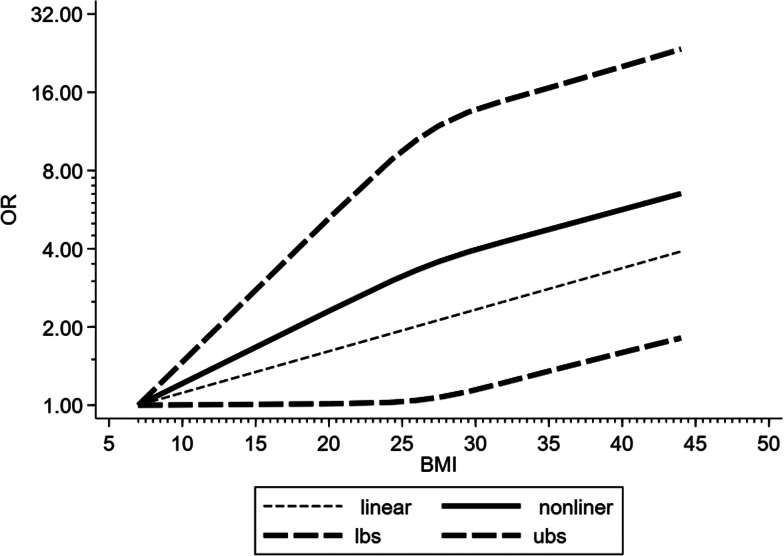
Dose−response relationship between BMI and risk of ICU admission for COVID-19 patients. Long-dashed black lines represent 95% CI. The vertical axes are on a log scale.

Six and five studies reported IMV treatment and ICU admission of COVID-19 patients with different BMI levels, respectively. Therefore, these studies were identified in the dose−response analysis. For every 5 kg/m^2^ increase in body mass index, the OR values of IMV use and ICU admission in COVID-19 patients were 1.16 (95%CI 1.10–1.23, *P* < 0.0001) and 1.2 (95% CI 1.11–1.30, *P* < 0.0001), respectively. And no non-linear dose−response relationship was detected (*P* 0.05) (Figs 6 and 7).

### Risk for mortality

**Fig. 8.**
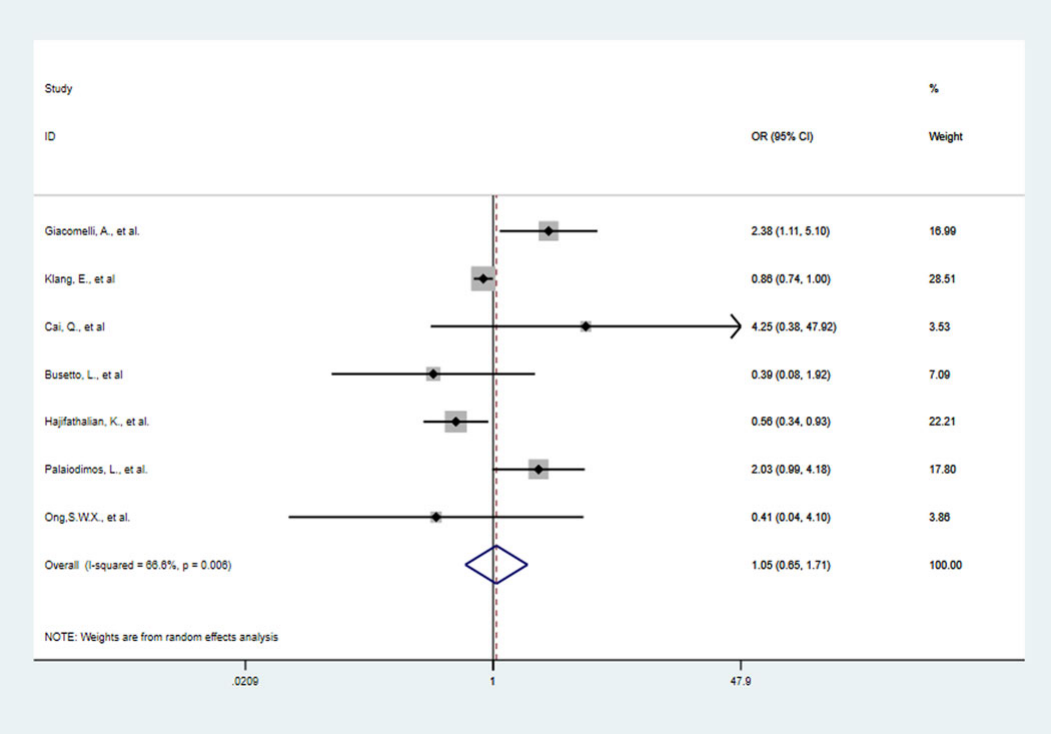
Forest plot of studies about association between obesity and risk of death.

The meta-analysis of seven studies involving 5175 patients showed that obesity was not associated with mortality in COVID-19 patients (OR 1.05, 95% CI 0.65–1.71, *P* = 0.84, *I*^2^ = 66.6%) (Fig. 8). The seven studies were subgroup analysed by different countries and obesity definitions. And in different countries and under different definitions of obesity, obesity was still not associated with mortality in COVID-19 patients. More details are hown in Table S5.

**Fig. 9.**
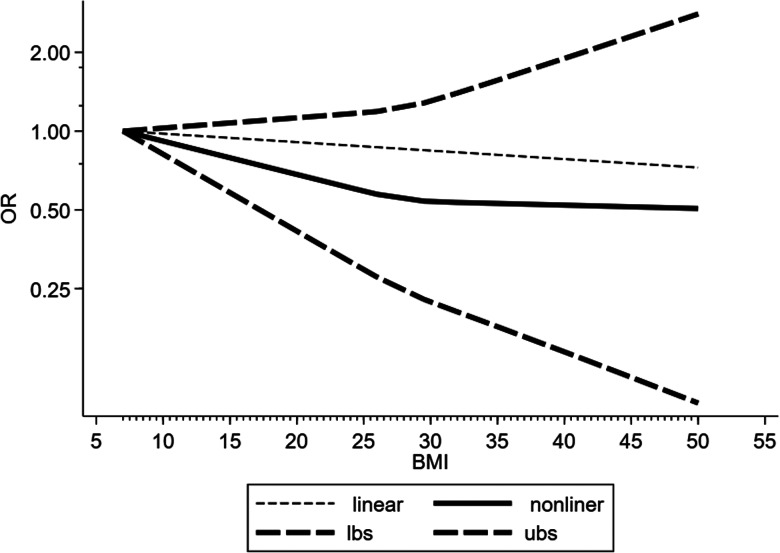
Dose−response relationship between BMI and risk of mortality in COVID-19 patients. Long-dashed black lines represent 95% CI. The vertical axes are on a log scale.

Four studies were included in the dose−response analysis. The results showed that the OR of death in COVID-19 patients per 5 kg/m^2^ increase in body mass index was 0.96 (95% CI 0.83–1.11, *P* = 0.62). And there may be a non-linear dose−response relationship (*P* < 0.05) (Fig. 9).

### Publication bias

The funnel plots of the prevalence, the funnel plots of the relationship between obesity and severe COVID-19 or mortality showed a symmetrical distribution, which mean no publication bias (Figs S5–S7).

## Discussion

In this meta-analysis among a total of 6081 patients with COVID-19, we found that the total prevalence of obesity was 30%. Obesity was associated with higher risks of severe COVID-19 (ICU admission and IMV management), while not death. And in dose−response analysis, an increase in BMI was associated with an increased risk of IMV use and ICU admission in COVID-19 patients, but not death, which suggests an ‘obesity survival paradox’ might exist for COVID-19.

COVID-19 has spread rapidly in the world and people of all ages can be infected by SARS-CoV-2. However, older people and people with pre-existing medical conditions, such as asthma, heart disease and diabetes, appear to be more vulnerable to becoming severely ill with the virus. Due to lack of information of BMI or waist circumference, which was not routinely measured especially in the early outbreak, the relationship of obesity and COVID-19 was reported late recently. Some investigations have reported that obesity might increase the risk of SARS-CoV-2 infection and affect the prognosis of patients with COVID-19 [12, 13]. Whereas some cohort studies with COVID-19 disease found obesity rates were generally reported as no higher than population-based estimates [14, 15]. A recent Italian report failed to mention obesity as a co-morbidity in admitted COVID-19 intensive care unit (ICU) patients [16]. In our analysis, the prevalence of obesity in patients with COVID-19 in China was 10.7%, in Europe was 18.3% and in USA was 41.4%. The prevalence is much lower than the estimated standardised prevalence of total obesity among the general adult residents, which is 17.8% in China, 20–23% in Europe and 42.4% in USA, respectively. So obesity is not associated with increased susceptibility for COVID-19 although sex- and age-adjusted population analysis of prevalence estimates is not available until now.

There is a growing body of research looking at the links between obesity and the severity of COVID-19 disease and death. Toussie *et al*. [23] reported that obesity increases the risk of hospitalisation (OR = 2.4) and IMV utilisation (OR = 2.38) for COVID-19 patients. Kalligeros *et al*. [24] found that obese patients are 1.92 times more likely to be admitted to ICU than non-obese patients with COVID-19. Giacomelli *et al*. [30] reported that COVID-19 patients with obesity had a 2.38 higher risk of mortality. Our meta-analysis of retrospective studies summarised nine studies and suggests that obesity is associated with the increased risk of severe COVID-19, which includes ICU admission and IMV (OR 1.79, 95% CI 1.52–2.11, *P* < 0.0001, *I*^2^ = 0%). COVID-19 patients with obesity had a 1.86 and 1.74 higher risk of ICU admission and IMV treatment, respectively. And COVID-19 patients had a 16% increased risk of using IMV and a 20% increased risk of ICU admission per 5 kg/m^2^ increase in BMI.

There are several underlying pathophysiological mechanisms associated with increased obesity and severe COVID-19 infection. First, angiotensin converting enzyme 2 (ACE2), which is a receptor for SARS-CoV-2 to enter into host cells [32], is expressed in fat cells [33]. And fatty infiltration of the lung is increased in obese patients [34]. Then in obese patients, expression of ACE2 in lung fat may influence the severity of COVID-19. Second, obesity increase the risk of cardiovascular disease and type 2 diabetes [35]. Metabolic diseases such as diabetes are co-morbidities of obesity. And cardiovascular disease and type 2 diabetes are risk factors for COVID-19 [5]. Then obese patients may increase their risk of severe COVID-19 by increasing their risk of co-morbidities. Third, obesity is a chronic inflammatory disease, may affect COVID-19 by enhancing local and systemic inflammatory responses through pro-inflammatory mediators produced by adipose tissue [36]. Finally, obesity can cause lung dysfunction, such as reduced functional residual capacity (FRC), reduced expiratory residual volume (ERV), decreased chest wall and decreased lung compliance [37, 38].

There is uncertainty about the association between obesity and pneumonia mortality. This present meta-analysis showed mortality risk was not different between obese COVID-19 patients and non-obese patients (OR 1.05, 95% CI 0.65–1.71, *P* = 0.84, *I*^2^ = 66.6%). Dose−response analysis also showed that an increase in body mass index was not associated with death in COVID-19 patients. So we did not find a survival disadvantage for obese patients with COVID-19, although the risk of severe COVID-19 was significantly increased. Previous studies have shown that obesity is associated with increased need of mechanical ventilation but not with mortality in critically ill patients from ICU [39]. Other studies reported that obese subjects with pneumonia or from ICU had lower mortality compared to non-obese subjects [19, 40, 41]. We propose two possible explanations for the result of our meta-analysis. First, obese patients have sufficient nutritional reserves to compensate for the stress of a critically ill condition. Second, obese people may be considered ‘at high risk’, so they might get more aggressive treatment [42]. However, due to limited data and confounding factors, further research is necessary to prove whether obesity is a risk factor for SARS-CoV-2 infection and whether there is an ‘obesity survival paradox’ in COVID-19. And more clinical studies need to be carried out to make the mechanism by which obesity affects the development and mortality of COVID-19 clear. Then these may be helpful to find appropriate immunotherapy for COVID-19 patient with obesity.

There are several limitations in our meta-analysis. First, data on BMI for patients with COVID-19 infections are very few, resulting in a limited number of studies included in the meta-analysis. Second, there are many confounders, such as age, co-existing other co-morbidities, physical activity. Because this meta-analysis is based on observational studies, more studies are required to confirm the results. Third, the included studies did not agree on the definition of obesity and the severity of the disease in COVID-19 patients.

## Conclusions

Our meta-analysis shows that the prevalence of obesity in COVID-19 patients is relatively high, and obesity is a risk factor for severe COVID-19, while not death. We understand the role of obesity in the progression of COVID-19. Then we can identify, treat and monitor COVID-19 patients with obesity early, based on BMI data that is easily available, to see who among these patients could benefit from aggressive treatment.

## Data Availability

The data that support the findings of this study are available in the supplementary material of this paper. And the code for the dose−response analysis is shown in supplementary file 2.

## Supplementary material

For supplementary material accompanying this paper visit http://dx.doi.org/10.1017/S0950268820003179. click here to view supplementary material
