# Supplementary material for: Obesity is associated with severe COVID-19 but not death: a dose−response meta-analysis
Source: Epidemiol Infect. 2021 Jan 5;149:e144. doi: 10.1017/S0950268820003179 (PMC8245341; doi:10.1017/S0950268820003179)
Supplement: Supplementary file 1 [file S0950268820003179sup001.zip › S0950268820003179sup001.docx]

Table S5. Subgroup analysis of studies about association between obesity and risk of mortality

| Group | No.of studies | OR | 95% CI | P-value |
| --- | --- | --- | --- | --- |
| Asia | 2 | 1.281 | 0.122-13.465 | 0.836 |
| Europe | 2 | 1.107 | 0.189-6.491 | 0.910 |
| USA | 3 | 0.923 | 0.549-1.551 | 0.762 |
| BMI≥25kg/m^2^ | 1 | 0.410 | 0.041-4.101 | 0.448 |
| BMI≥28kg/m^2^ | 1 | 4.250 | 0.377-47.925 | 0.242 |
| BMI≥30kg/m^2^ | 4 | 0.883 | 0.520-1.501 | 0.646 |
| BMI≥35kg/m^2^ | 1 | 2.033 | 0.989-4.181 | 0.054 |
| overall | 7 | 1.052 | 0.647-1.710 | 0.838 |
