# Supplementary material for: Obesity is associated with severe COVID-19 but not death: a dose−response meta-analysis
Source: Epidemiol Infect. 2021 Jan 5;149:e144. doi: 10.1017/S0950268820003179 (PMC8245341; doi:10.1017/S0950268820003179)
Supplement: Supplementary file 1 [file S0950268820003179sup001.zip › S0950268820003179sup010.docx]

Code for the dose-response analysis

ssc install glst

bysort id: gen bmic=bmi-bmi[1]

mkspline bmics=bmic, nk(3) cubic displayknots

mkspline bmis=bmi, nk(3) cubic displayknots

mvmeta_make glst logor bmics1 bmics2, cov (n case) se(selogor) pfirst (id studyt)saving(ssest_spline) replace by (id) names (b V)

preserve

use ssest_spline, clear

mvmeta b V, mm i2

testparm bbmics2

capture estimates save mvmeta, replace

restore

estimates use mvmeta

su bmis1 bmis2 if bmi==float(7)

predictnl logor_sp=_b[bbmics1]*(bmis1-7)+_b[bbmics2]*(bmis2-0),ci(los his)

gen ors=exp(logor_sp)

gen lbs=exp(los)

gen ubs=exp(his)

glst logor bmic, cov(n case) se(selogor) pfirst(id studyt) ts(r)

lincom bmic*5,eform

predictnl logor_l=_b[bmic]*(bmi-7), ci(lo hi)

gen orl=exp(logor_l)

gen lblin=exp(lo)

gen ublin=exp(hi)

lincom bmic*5,eform

twoway (line orl bmi, sort lc(black)lp(-))(line ors lbs ubs bmi, sort lw(thick thick thick) lc(black black black) lp(l longdash longdash)), yscale(log) ytitle(OR) xtitle(BMI) scheme(s1mono) xlabel(7(1)50) xmtick(7(.5)50) ymtick(1(.5)8) ylabel(1 2 4 8 16, format(%3.2fc) angle(horiz))legend(label(1 linear) label(2 nonliner) )plotregion(style(none))

twoway (line orl bmi, sort  lc(black) lp(-) ) (line ors lbs ubs bmi, sort lw(thick thick thick) lc(black black black) lp(l longdash longdash)) , yscale(log) ytitle(OR) xtitle(BMI) scheme(s1mono) xlabel(7(1)50) xmtick(7(.5)50) ymtick(1(.5)8) ylabel(1 2 4 8 , format(%3.2fc) angle(horiz)) legend(label(1 Linear Model)  label(2 Spline Model)) order(1 2) ring(0) pos(11) col(1) ) plotregion(style(none))

order(1 2) ring(0) pos(11) col(1) plotregion (style(none))

levelsof bmi， local(level)

xblcbmis*,c(bmi)at(‘r(levels)’)ref(0)eform
