# Supplementary material for: Obesity is associated with severe COVID-19 but not death: a dose−response meta-analysis
Source: Epidemiol Infect. 2021 Jan 5;149:e144. doi: 10.1017/S0950268820003179 (PMC8245341; doi:10.1017/S0950268820003179)
Supplement: Supplementary file 1 [file S0950268820003179sup001.zip › S0950268820003179sup011.docx]

| id | author | BMI category  (kg/m^2^) | BMI | Using IMV(n) | Control  (n) | Total(n) |
| --- | --- | --- | --- | --- | --- | --- |
| 1 | Hur, K., et al. | <30 | 20 | 55 | 172 | 227 |
| 1 | Hur, K., et al. | 30-39.99 | 35 | 58 | 129 | 187 |
| 1 | Hur, K., et al. | ≥40 | 50 | 25 | 47 | 72 |
| 2 | Toussie, D., et al. | <25 | 21 | 3 | 19 | 22 |
| 2 | Toussie, D., et al. | 26-30 | 28 | 5 | 38 | 43 |
| 2 | Toussie, D., et al. | 31-40 | 35.5 | 12 | 46 | 58 |
| 2 | Toussie, D., et al. | >40 | 49 | 8 | 14 | 22 |
| 3 | Cai, Q., et al | <23.9 | 19.9 | 16 | 203 | 219 |
| 3 | Cai, Q., et al | 24-27.9 | 26 | 14 | 109 | 123 |
| 3 | Cai, Q., et al | ≥28 | 31.9 | 5 | 36 | 41 |
| 4 | Busetto, L., et al | <25 | 20 | 2 | 30 | 32 |
| 4 | Busetto, L., et al | 25-30 | 27.5 | 5 | 26 | 31 |
| 4 | Busetto, L., et al | ≥30 | 35 | 2 | 27 | 29 |
| 5 | Hajifathalian, K., et al. | <18.5 | 7 | 3 | 25 | 28 |
| 5 | Hajifathalian, K., et al. | 18.5-30 | 24.3 | 99 | 366 | 465 |
| 5 | Hajifathalian, K., et al. | >30 | 41.5 | 91 | 186 | 277 |
| 6 | Palaiodimos, L., et al. | <25 | 16 | 7 | 31 | 38 |
| 6 | Palaiodimos, L., et al. | 25-34 | 29.5 | 19 | 97 | 116 |
| 6 | Palaiodimos, L., et al. | ≥35 | 44 | 16 | 30 | 46 |

Table S1. The characteristics of the six studies included in the dose-response meta-analysis about BMI and OR of IMV using
