# Supplementary material for: Obesity is associated with severe COVID-19 but not death: a dose−response meta-analysis
Source: Epidemiol Infect. 2021 Jan 5;149:e144. doi: 10.1017/S0950268820003179 (PMC8245341; doi:10.1017/S0950268820003179)
Supplement: Supplementary file 1 [file S0950268820003179sup001.zip › S0950268820003179sup012.docx]

Table S2. The characteristics of the five studies included in the dose-response meta-analysis about BMI and OR of ICU admission.

| id | author | BMI category  (kg/m^2^) | BMI | ICU admission  (n) | Control  (n) | Total(n) |
| --- | --- | --- | --- | --- | --- | --- |
| 1 | Kalligeros, M., et al. | <25 | 20.1 | 5 | 14 | 19 |
| 1 | Kalligeros, M., et al. | 25-29.9 | 27.5 | 14 | 21 | 35 |
| 1 | Kalligeros, M., et al. | 30-34.9 | 32.5 | 11 | 11 | 22 |
| 1 | Kalligeros, M., et al. | ≥35 | 39.9 | 14 | 13 | 27 |
| 2 | Cai, Q., et al | <23.9 | 19.9 | 16 | 203 | 219 |
| 2 | Cai, Q., et al | 24-27.9 | 26 | 14 | 109 | 123 |
| 2 | Cai, Q., et al | ≥28 | 31.9 | 5 | 36 | 41 |
| 3 | Busetto, L., et al | <25 | 20 | 2 | 30 | 32 |
| 3 | Busetto, L., et al | 25-30 | 27.5 | 7 | 24 | 31 |
| 3 | Busetto, L., et al | ≥30 | 35 | 7 | 22 | 29 |
| 4 | Hajifathalian, K., et al. | <18.5 | 7 | 5 | 23 | 28 |
| 4 | Hajifathalian, K., et al. | 18.5-30 | 24.3 | 99 | 366 | 465 |
| 4 | Hajifathalian, K., et al. | >30 | 41.5 | 92 | 185 | 277 |
| 5 | Palaiodimos, L., et al. | <25 | 16 | 3 | 35 | 38 |
| 5 | Palaiodimos, L., et al. | 25-34 | 29.5 | 18 | 98 | 116 |
| 5 | Palaiodimos, L., et al. | ≥35 | 44 | 11 | 35 | 46 |
