# Supplementary material for: Obesity is associated with severe COVID-19 but not death: a dose−response meta-analysis
Source: Epidemiol Infect. 2021 Jan 5;149:e144. doi: 10.1017/S0950268820003179 (PMC8245341; doi:10.1017/S0950268820003179)
Supplement: Supplementary file 1 [file S0950268820003179sup001.zip › S0950268820003179sup013.docx]

Table S3. The characteristics of the four studies included in the dose-response meta-analysis about BMI and OR of mortality.

| id | author | BMI category  (kg/m^2^) | BMI | Death  (n) | Control  (n) | Total(n) |
| --- | --- | --- | --- | --- | --- | --- |
| 1 | Klang, E., et al | <30 | 20 | 752 | 1423 | 2175 |
| 1 | Klang, E., et al | 30-40 | 35 | 290 | 667 | 957 |
| 1 | Klang, E., et al | ≥40 | 50 | 94 | 180 | 274 |
| 2 | Cai, Q., et al | <23.9 | 19.9 | 1 | 218 | 219 |
| 2 | Cai, Q., et al | 24-27.9 | 26 | 1 | 122 | 123 |
| 2 | Cai, Q., et al | ≥28 | 31.9 | 1 | 40 | 41 |
| 3 | Hajifathalian, K., et al. | <18.5 | 7 | 9 | 19 | 28 |
| 3 | Hajifathalian, K., et al. | 18.5-30 | 24.3 | 57 | 399 | 456 |
| 3 | Hajifathalian, K., et al. | >30 | 41.5 | 22 | 255 | 277 |
| 4 | Palaiodimos, L., et al. | <25 | 16 | 12 | 26 | 38 |
| 4 | Palaiodimos, L., et al. | 25-34 | 29.5 | 20 | 96 | 116 |
| 4 | Palaiodimos, L., et al. | ≥35 | 44 | 16 | 20 | 36 |
