# Supplementary material for: Obesity is associated with severe COVID-19 but not death: a dose−response meta-analysis
Source: Epidemiol Infect. 2021 Jan 5;149:e144. doi: 10.1017/S0950268820003179 (PMC8245341; doi:10.1017/S0950268820003179)
Supplement: Supplementary file 1 [file S0950268820003179sup001.zip › S0950268820003179sup014.docx]

Table S4. Subgroup analysis of prevalence.

| Group | No.of studies | ES | 95% CI | P-value |
| --- | --- | --- | --- | --- |
| Asia | 2 | 0.109 | 0.081-0.138 | 0 |
| Europe | 3 | 0.183 | 0.086-0.280 | 0 |
| USA | 6 | 0.414 | 0.339-0.490 | 0 |
| BMI≥28kg/m^2^ | 1 | 0.107 | 0.076-0.138 | 0 |
| BMI≥30kg/m^2^ | 9 | 0.329 | 0.237-0.421 | 0 |
| BMI≥35kg/m^2^ | 1 | 0.230 | 0.172-0.288 | 0 |
| overall | 11 | 0.300 | 0.210-0.389 | 0 |
