# Supplementary figures and images for: Obesity is associated with severe COVID-19 but not death: a dose−response meta-analysis
Source: Epidemiol Infect. 2021 Jan 5;149:e144. doi: 10.1017/S0950268820003179 (PMC8245341; doi:10.1017/S0950268820003179)

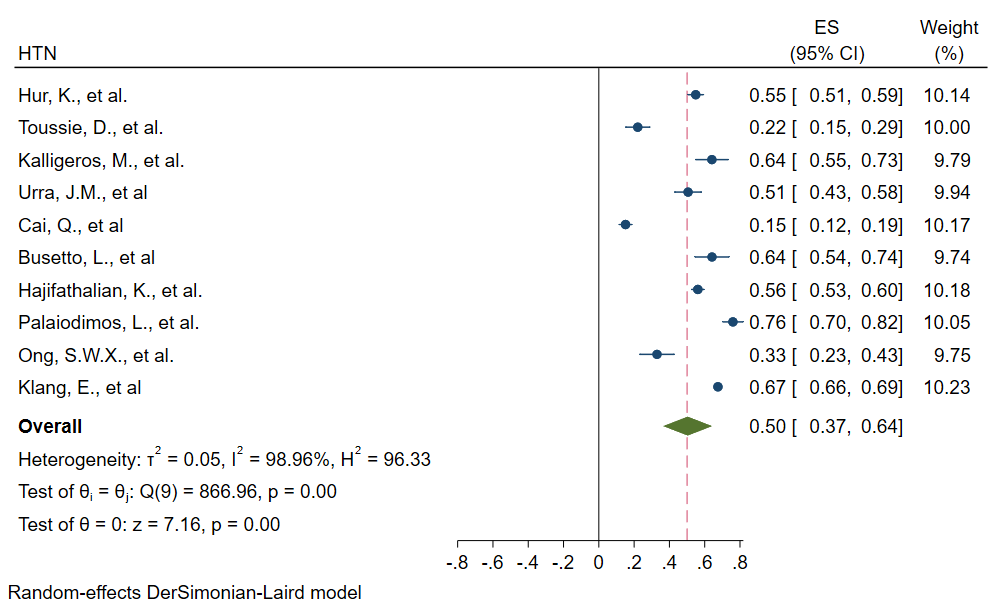

Supplement: Supplementary file 1 [file S0950268820003179sup001.zip › S0950268820003179sup002.png]

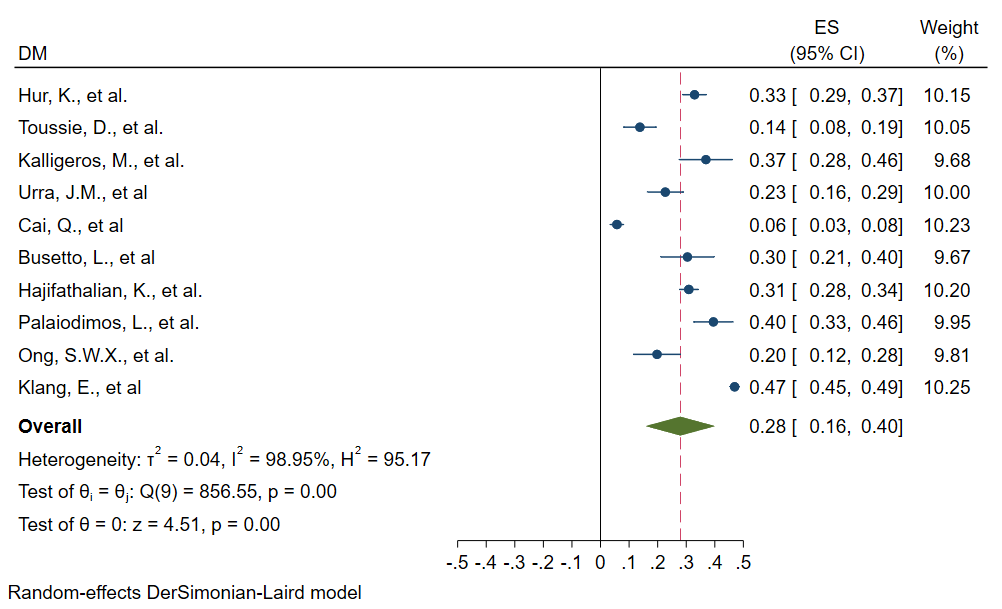

Supplement: Supplementary file 1 [file S0950268820003179sup001.zip › S0950268820003179sup003.png]

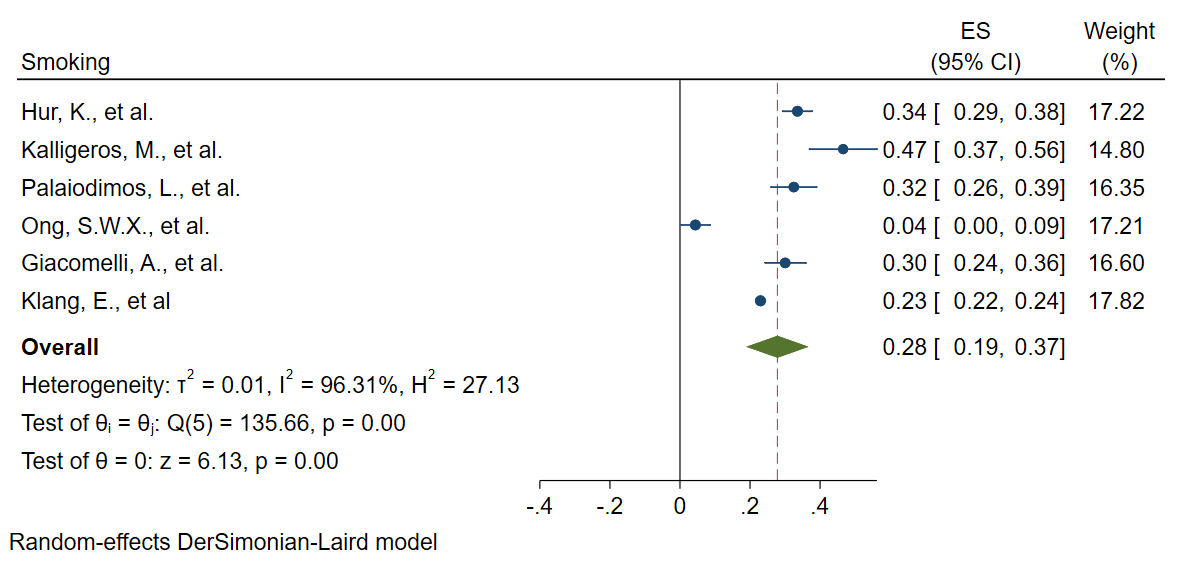

Supplement: Supplementary file 1 [file S0950268820003179sup001.zip › S0950268820003179sup004.png]

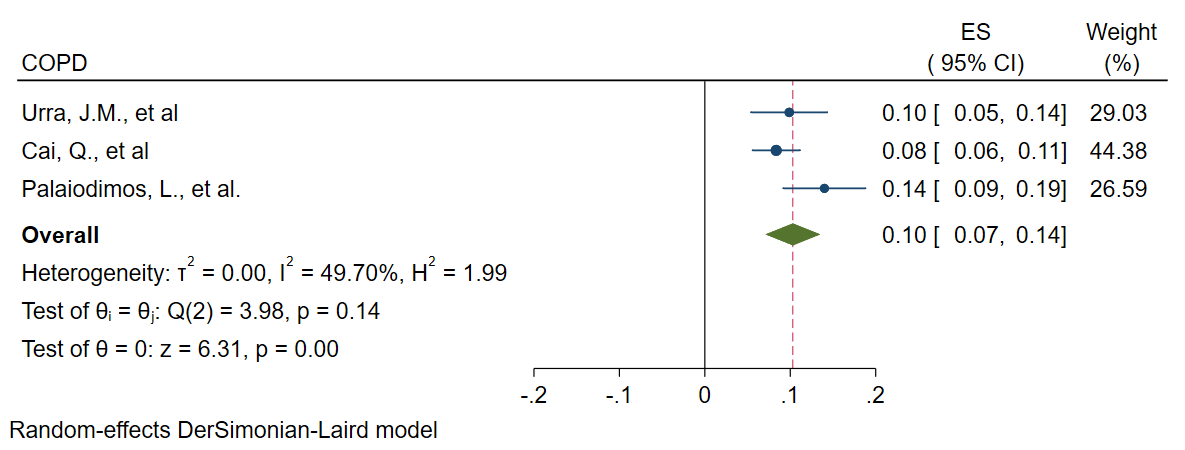

Supplement: Supplementary file 1 [file S0950268820003179sup001.zip › S0950268820003179sup005.png]

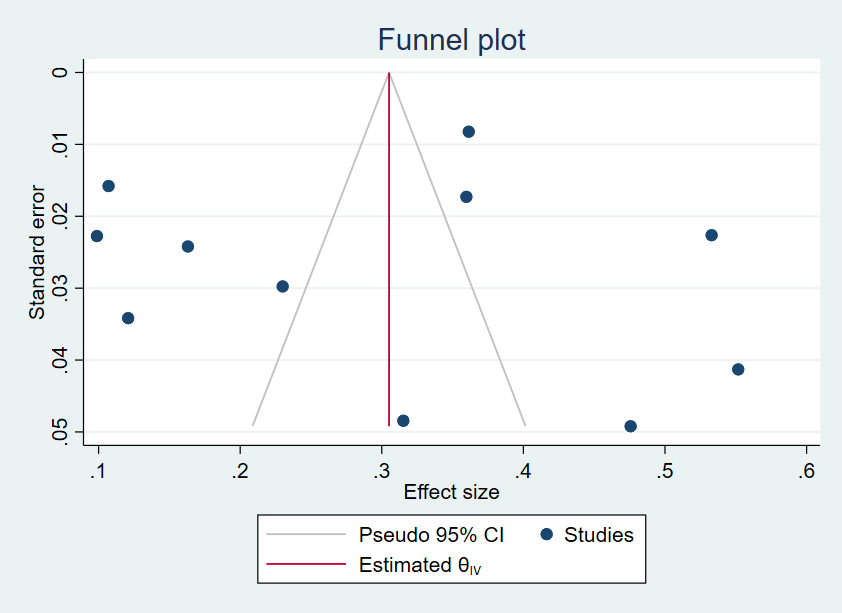

Supplement: Supplementary file 1 [file S0950268820003179sup001.zip › S0950268820003179sup006.png]

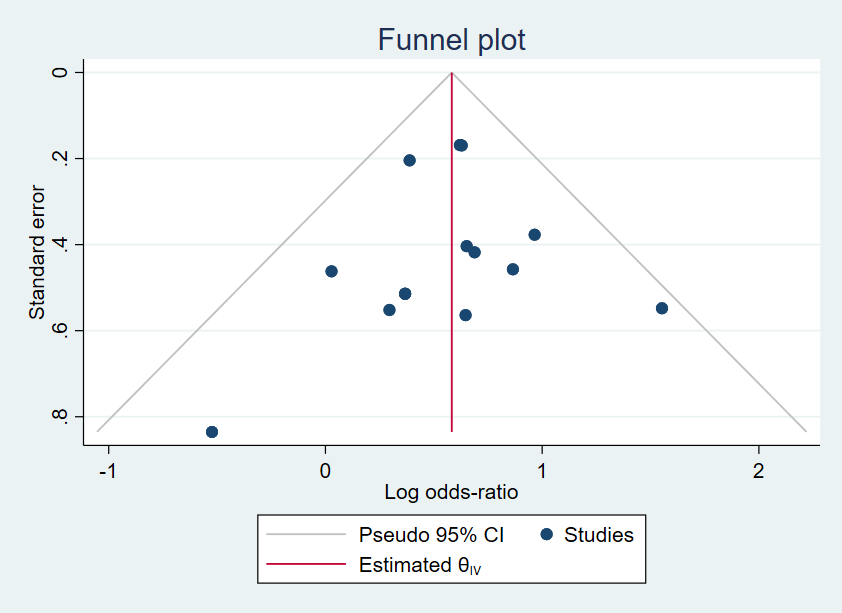

Supplement: Supplementary file 1 [file S0950268820003179sup001.zip › S0950268820003179sup007.png]

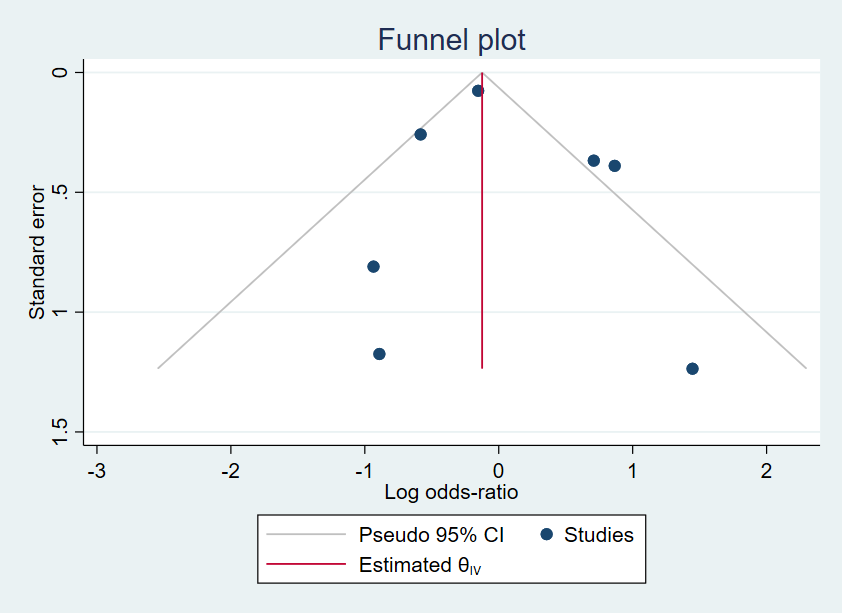

Supplement: Supplementary file 1 [file S0950268820003179sup001.zip › S0950268820003179sup008.png]
